# The effect of posttransplant cyclophosphamide on ocular graft-versus-host disease

**DOI:** 10.1038/s41409-025-02628-9

**Published:** 2025-05-30

**Authors:** Leonie Menghesha, Volkan Tahmaz, Udo Holtick, Christof Scheid, Michael E. Stern, Philipp Steven

**Affiliations:** 1 Competence Center for Ocular GVHD, Clinic I of Internal Medicine and Department of Ophthalmology, University of Cologne, Cologne, Germany; 2 Department of Ophthalmology, St. Elisabeth Hospital Hohenlind, Cologne, Germany; 3 Clinic I of Internal Medicine, Center of Integrated Oncology Köln Bonn, University of Cologne, Faculty of Medicine and University Hospital Cologne, University of Cologne, Cologne, Germany

**Keywords:** Stem-cell research, Haematopoietic stem cells

## Introduction

Recently, it was recommended that a 3-drug combination of post-transplant cyclophosphamide (PT-Cy), tacrolimus (Tac) and mycophenolate mofetil should be used not only for mismatched transplants but also for closely matched donors. Regarding PT-Cy, it has been shown that a higher 1-year graft-versus-host disease (GVHD)-free and relapse-free survival was achieved compared to standard GVHD prophylaxis with an exclusive calcineurin inhibitor such as Tac and methotrexate [1]. This progress has the potential to improve the general outcome of allogeneic hematopoietic stem cell transplantation (aHCT). As 40–60% of patients develop chronic ocular GVHD (oGVHD), which is one of the main reasons for reduction of quality-of-life [2] after aHCT, strategies to prevent or reduce severity of chronic oGVHD are urgently needed. Nevertheless, limited data is available regarding the impact of PT-Cy on the eye.

## Methods

This single-centre retrospective study relied on data acquired at the Departments of Ophthalmology and Internal Medicine, University Hospital of Cologne, Germany between May 2011 and January 2023. Regular ophthalmological consultation, independently of the occurrence of symptoms, are standard of care at our clinic.

Medical records of the first 63 consecutive patients (mean age 48.4 years ± 15.14) who received a GVHD prophylaxis scheme with PT-Cy (Cyclophosphamide 50 mg/kg/day on days 3 and 4) were screened. Epidemiological data, including age, gender, transplant date, main diagnosis, health status after conditioning, conditioning regime, type of donor relationship, occurrence of acute and chronic GVHD, including NIH and ICCGVHD Grading for chronic oGVHD were collected. Ophthalmological examinations from first and last visit after aHCT included best spectacle-corrected visual acuity (BSCVA), slit lamp bio-microscopy, Schirmer’s test I, corneal aesthesiometer (Cochet-Bonnet), tear-film-break-up-time, measurement of intraocular pressure, grading of corneal and conjunctival staining with the Oxford Schema. Subjective symptoms were assessed with a symptom-oriented questionnaire (‘Ocular Surface Disease Index’, OSDI^®^). Only exclusion criterium was a lack of ophthalmological consultations after aHCT (*n* = 26). A total of 37 patients qualified for further analysis.

Descriptive data were collected and analysed by SPSS (version 28.0 for Windows; SPSS, Inc., Chicago, IL, USA). The BSCVA was converted to the logarithm of Minimum Angle of Resolution (logMAR). Depending on normal distribution of the interval-scaled parameters we analysed, we conducted the Chi-Square test and the unequal variances t-test. The level of significance was defined as *p* < 0.05.

## Results

Medical records of 63 consecutive patients (mean age 48.4 ± 15.14 years) who received PT-Cy were screened. Peripheral blood was the stem cell source for all patients. While 42 patients (66.6%) suffered from acute GVHD, 21 patients (33.3%) were affected by chronic GVHD during the observation period. Overall severity of cGVHD was NIH grade I (range 0–III). In acute GVHD, the skin (*n* = 35) was predominantly affected, whereas the eyes (*n* = 15) were the most frequent localisation of chronic GVHD. One patient had severe cGHVD of the lung. A total of 37 patients, who underwent at least two routine ophthalmological examinations after aHCT, were eligible for further analysis. Of these patients, 18 (48.6%) had 10/10 HLA matching, 7 (18.9%) had 9/10 matching and 12 (32.4%) had 5/10 matching.

Patients with only one or no ophthalmological exam were excluded, as the necessary ophthalmological data to contribute to the analysis were missing. Patient characteristics are shown in Table 1.

**Table 1:** Patient characteristics including GVHD grading.

Patient characteristics	Total	No chronic oGVHD	Chronic oGVHD	
Demographic variable	*n* (%)	*n* (%)	*n* (%)	
	37 (100.0)	22 (59.5)	15 (40.5)	
Gender				
Male	23 (62.2)	15 (68.2)	8 (53.3)	
Female	14 (37.8)	7 (31.8)	7 (46.7)	
Mean age (±SD) in years	45.38 (±15.27)	40.36 (±15.63)	52.73 (±11.65)	
Primary Disease				
Acute lymphoblastic leukaemia (ALL)	2 (5.4)	1 (4.5)	1 (4.5)	
Acute myelogenous leukaemia (AML)	8 (21.6)	7 (31.8)	1 (4.5)	
Chronic lymphocytic leukaemia (CLL)	6 (16.2)	2 (9.1)	4 (26.7)	
Chronic myelogenous leukaemia (CML)	3 (8.1)	1 (4.5)	2 (13.3)	
Myelodysplastic syndrome (MDS)	2 (5.4)	0 (0.0)	2 (13.3)	
Lymphoma (all subtypes)	15 (40.5)	10 (45.5)	5 (33.3)	
Blastic plasmacytoid dendritic cell neoplasm (BPDCN)	1 (2.7)	1 (4.5)	0 (0.0)	
Donor type				
Related donor	20 (54.1)	11 (50.0)	9 (60.0)	
Unrelated donor	17 (45.9)	11 (50.0)	6 (40.0)	
Conditioning regimen				
Fludarabine, busulfan	22 (59.5)	12 (54.5)	10 (66.7)	
Fludarabine, treosulfan	8 (21.6)	5 (22.7)	3 (20.0)	
FLAMSA, TBI 12 Gy	3 (8.1)	2 (9.1)	1 (6.7)	
Other	4 (10.8)	3 (13.6)	1 (6.7)	
GVHD prophylaxis				
PT-Cy, CSA, MMF	15 (40.5)	10 (45.5)	5 (33.3)	
PT-Cy, CSA, everolimus	1 (2.7)	1 (4.5)	0 (0.0)	
PT-Cy, everolimus, MMF	1 (2.7)	1 (4.5)	0 (0.0)	
PT-Cy, tacrolimus, MMF	5 (13.5)	2 (9.1)	3 (20.0)	
PT-Cy, MMF	1 (2.7)	0 (0.0)	1 (6.7)	
PT-Cy, everolimus	13 (35.1)	7 (31.8)	6 (40.0)	
PT-Cy, everolimus, MMF, ATG	1 (2.7)	1 (2.7)	0 (0.0)	
GVHD				
Acute skin GVHD	24 (64.9)	13 (59.1)	11 (7.3)	
Acute gut GVHD	4 (10.8)	1 (4.5)	3 (20.0)	
Chronic skin GVHD	5 (13.5)	3 (13.6)	2 (13.3)	
NIH grade I	4 (10.8)	3 (13.6)	1 (6.7)	
NIH grade II	1 (2.7)	0 (0)	1 (6.7)	
Chronic gut GVHD	3 (8.1)	2 (9.1)	1 (6.7)	
NIH grade I	2 (5.4)	2 (9.1)	0 (0)	
NIH grade II	1 (2.7)	0 (0)	1 (6.7)	
Non-relapse mortality	8 (21.6)	5 (22.7)	3 (20.0)	
Chronic oGVHD				
**NIH grading**	**No chronic oGVHD**	**Grade I**	**Grade II**	**Grade III**
	*n* (%)	*n* (%)	*n* (%)	*n* (%)
*n* = 15	0 (0.0)	3 (20.0)	4 (26.7)	8 (53.3)
**ICCGVHD**	**None (0–4)**	**Mild (5–8)**		**Severe (9–11)**
	*n* (%)	*n* (%)		*n* (%)
*n* = 15	0 (0)	10 (66.7)		5 (33.3)

### Ocular GVHD

Fifteen of these 37 patients (40.54%) developed chronic oGVHD. Acute oGVHD was present in three patients (18.75%); two of these developed chronic oGVHD consecutively. The distribution regarding NIH and ICCGVHD grading is shown in Table 1.

Comparing NIH grading results with data from the previously published Cologne oGVHD cohort [3], differences were found. In the previously analysed cohort 15% of the patients had oGVHD NIH I, 22% NIH II and 63% NIH III.

### Adverse environmental stress (AES)

Adverse environmental conditions during aHCT influence the development of oGVHD [3]. We divided patients into two groups depending on their transplant date. Of all patients transplanted in summer (*n* = 16), a total of 5 patients (31.2%) developed chronic oGVHD (ICCGVHD: mild-moderate: *n* = 3, severe: *n* = 2). Of all patients transplanted in winter (October–February, *n* = 21), a total of 10 patients (47.6%) developed chronic oGVHD (ICCGVHD: mild-moderate *n* = 7, severe *n* = 3).

### Ophthalmological examination

Ophthalmological findings of 37 patients without (*n* = 22) and with (*n* = 15) chronic oGVHD were assessed. There are statistically significant differences between patients with and without chronic oGVHD for Schirmer’s test I (OD *p* = 0.018, OS *p* = 0.045), Oxford grade for corneal fluorescein staining (OD *p* = 0.002, OS *p* < 0.001) and OSDI® score (*p* = 0.002).

## Discussion

Primary aim of this analysis was to evaluate whether PT-Cy has an impact on the development and severity of chronic oGVHD. So far, there is only limited data available on how systemic GVHD prophylaxis affects oGVHD. Potential reasons are that ocular exams were not part of PT-Cy studies or post-transplant eye care is performed without close collaboration with transplant centres. The occurrence of oGVHD after PT-Cy was only mentioned in three patients in a retrospective analysis, which investigated the use of PT-Cy after bone marrow transplantation in nine patients with non-malignant haematological conditions [4]. Proportion of patients developing chronic oGVHD who received PT-Cy (40.54%) was comparable to the prevalence of chronic oGVHD in other cohorts [5] and in our Cologne cohort of 1-year survivors (*n* = 233, 48% chronic oGVHD) [3]. Compared to these cohorts, the overall severity of chronic oGVHD seems to be lower in patients receiving PT-Cy (NIH grade III: 46.7% vs 63%). Although there is no clinically significant reduction of chronic oGVHD prevalence in our cohort, the reduced overall severity could be PT-Cy-associated. The triggering effect of peri-transplant AES on the development of chronic oGVHD is known [3, 6]. To investigate whether PT-Cy would diminish this environmental effect, we distributed the patients into two groups depending on the time point of transplantation. Comparable to our previous study, we saw a higher number of patients developing chronic oGVHD with increased severity if aHCT was performed in winter. This implicates that PT-Cy does not countervail the effect of AES on the eyes. Regarding other risk factors for the development of chronic oGVHD, further studies with higher patient numbers are necessary. Randomised trials show that PT-Cy reduces moderate to severe cGVHD but not the overall incidence, suggesting a shift in severity rather than full prevention. This may explain the unchanged frequency of oGVHD, possibly influenced by environmental factors such as AES. Although overall cGVHD rates may be similar, PT-Cy has been associated with lower rates of steroid-refractory GVHD and a reduced need for prolonged or intensive immunosuppression, factors that may also influence the severity and management of oGVHD [7]. Future prospective clinical trials on PT-Cy or other prophylactic strategies should include regular eye examinations, including screenings prior to aHCT to identify patients with pre-existing dry eye disease and meibomian gland dysfunction, as these might be at higher risk for the development of oGVHD [8]. GVHD prophylaxis with PT-Cy demonstrates only a limited effect on the development of chronic oGVHD. This supports the hypothesis that pathogenesis of oGVHD occurs at least partially independent from systemic GVHD. Limitations of the present study are its size, mixed collective and retrospective design. In summary, the development of chronic oGVHD might be in part independent from the systemic GVHD prophylaxis. In contrast, more emphasis should be put on independent risk factors such as AES.

## Data Availability

The datasets generated during and/or analysed during the current study are not publicly available due security and ongoing research.
